# PD-L1 and EBV LMP1 expressions in classic Hodgkin lymphomas and its correlation with clinicopathological parameters and prognosis

**DOI:** 10.55730/1300-0144.5403

**Published:** 2022-05-12

**Authors:** Süleyman ÖZDEMİR, Özlem TON, Fevziye KABUKCUOĞLU

**Affiliations:** Department of Pathology, University of Health Sciences, Şişli Hamidiye Etfal Health Practice and Research Center, İstanbul, Turkey

**Keywords:** Hodgkin lymphoma, programmed death-ligand 1, Ebstein-Barr virus, prognosis

## Abstract

**Background/aim:**

Programmed death pathway leads to T cell anergy. Wide range of malignancies take advantage of this pathway by programmed death-ligand 1 (PD-L1) expression either on neoplastic cells or on the nonneoplastic cells of tumour microenvironment. New therapeutic approaches have been directed against this pathway. We studied PD-L1 expression on both neoplastic Hodgkin and Reed–Sternberg (HRS) cells and cells of tumour microenvironment in classic Hodgkin lymphoma (CHL) patients and compared it with Ebstein–Barr virus (EBV) positivity, clinical data, and survival rates.

**Materials and methods:**

Lymph node excision materials of 56 CHL patients diagnosed between 2007 and 2017 were included in this retrospective study. PD-L1 expression of HRS cells and tumour microenvironment cells were evaluated by immunohistochemical assay. Staining intensity and rate of the PD-L1 expressions were estimated. EBV was examined by immunohistochemistry for latent membrane protein 1 (LMP1) antibody. Clinical data of 39 patients and survival data of 34 patients were compared with PD-L1 expressions on tumour cells.

**Results:**

PD-L1 expression was present in HRS cells in 89.2% of the cases. There was more than 20% of PD-L1 expression in cells of tumour microenvironment in all the cases. PD-L1 positivity did not show statistically significant difference according to EBV expression, clinical parameters, and prognosis.

**Conclusion:**

Previous studies showed inconsistent rates for PD-L1 prevalence (20%–95.7%) in CHL patients due to differences in the study methods. Although high prevalence of PD-L1 positivity was found in majority of them, there was no statistically significant difference between PD-L1 positivity on HRS cells and EBV expression, clinical parameters, and prognosis. This high prevalence in patients with various clinical properties makes PD-L1 a potential target for new emerging immunotherapies for CHL.

## 1. Introduction

Antigen presentation to T-Helper (Th) cells is achieved by the interaction between major histocompatibility complex (MHC) protein on the surface of antigen presenting cells (APCs) and T-cell receptor (TCR) on the surface of Th cells. It is controlled by the programmed death (PD) pathway, which consists of the programmed death-ligand 1 (PD-L1) protein on the surface of APC, and its receptor programmed cell death protein 1 (PD1) on the surface of T cells. PD-L1 activates PD1, which inhibits TCR expression on the T cells and stops inflammation [1].

Malignancies can also escape from cellular immunity due to PD-L1 expressed either by the neoplastic cells themselves or by the reactive cells in tumour microenvironment. For this reason, there are many attempts for a monoclonal antibody therapy against the PD pathway. Such molecules are currently being used against other malignancies [1–3].

Classic Hodgkin lymphoma (CHL) derives from the germinal centre B cells that have lost their ability to synthesize immunoglobulins. Neoplastic Hodgkin and Reed–Sternberg (HRS) cells constitute about 1% of the total tumour area; the rest is reactive cells consisting of lymphocytes, macrophages, eosinophils, neutrophils, and fibroblasts [4]. Although it depends on histological subtype and geographical region, nearly half of the cases are Ebstein–Barr virus (EBV)-positive. Studies show that latent membrane protein 1 (LMP1), a viral protein, has a role in the pathogenesis [5–7]. Recently, two anti-PD1 molecules, nivolumab and pembrolizumab, are approved by the Food and Drug Administration (FDA) for the CHL patients who have relapsed disease after bone marrow transplantation [8,9].

There are several articles in the literature that study expression rates alone or with clinical correlation. However, there is variability in both expression rates and clinical correlation. The aim of this study is to determine the PD-L1 and EBV LMP1 expressions in classic Hodgkin lymphoma and its correlation with clinicopathological parameters and prognosis.

## 2. Material and method

### 2.1. Patients

Lymph node excisions of 56 patients diagnosed with CHL between 2007 and 2017 in our pathology department were included in this retrospective study. Clinical data (age, sex, stage on diagnosis, B symptoms, splenic or bone marrow involvement, extranodal involvement) of 39 patients were found and prognostic data (overall monitored time, overall disease-free survival time, relapse, death from disease) of 34 patients could be obtained from hospital records (Table 1). They had got either standard brentuximab vedotin, doxorubicin, vinblastine, dacarbazine (ABVD) or bleomycin, etoposide, doxorubicin hydrochloride (adriamycin), cyclophosphamide, vincristine (oncovin), procarbazine and prednisone (BEACOPP) protocols with or without radiotherapy according to current protocols at the time of diagnosis.

### 2.2. Immunhistochemistry

Three-micrometre thick slices of paraffin-embedded blocks on positive charged slides were prepared. Immunohistochemistry staining was performed on Leica Bond III autostainer using PD-L1 (clone: E1L3N, Cell Signaling Technologies, USA) and EBV LMP1 (clone: MRQ-47, Cell Marque, USA) with the 3,3′-Diaminobenzidine (DAB) peroxidase method.

**Figure 1:**
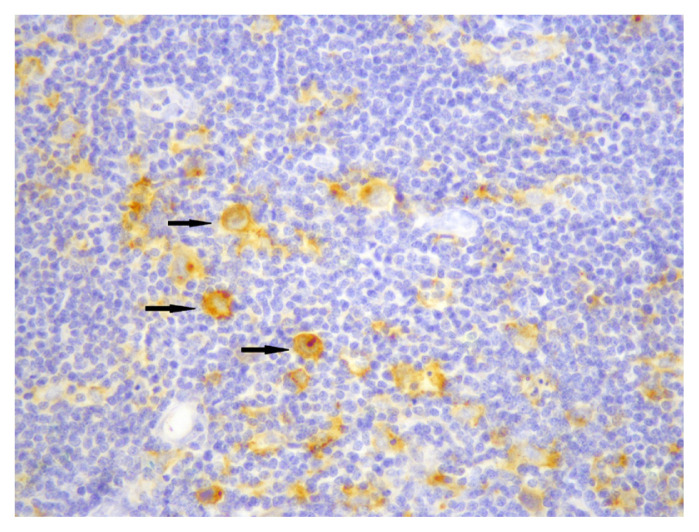
Weak (+1) membranous staining for PD-L1 in HRS cells (arrows) (×400).

**Figure 2:**
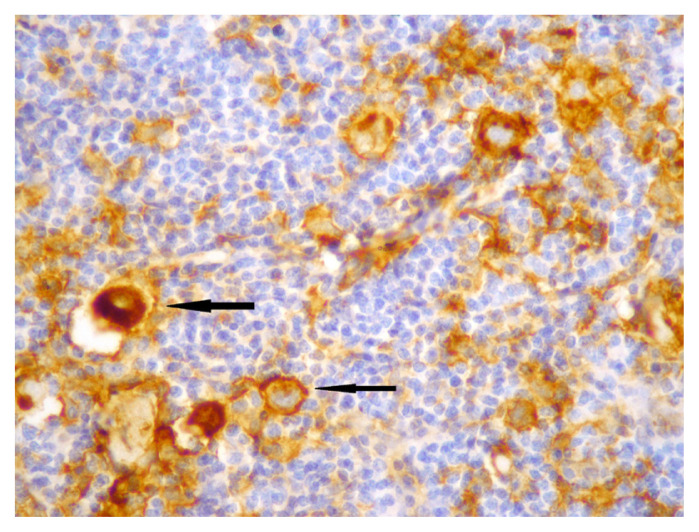
Moderate (+2) membranous staining for PD-L1 in HRS cells (arrows) (×400).

**Figure 3:**
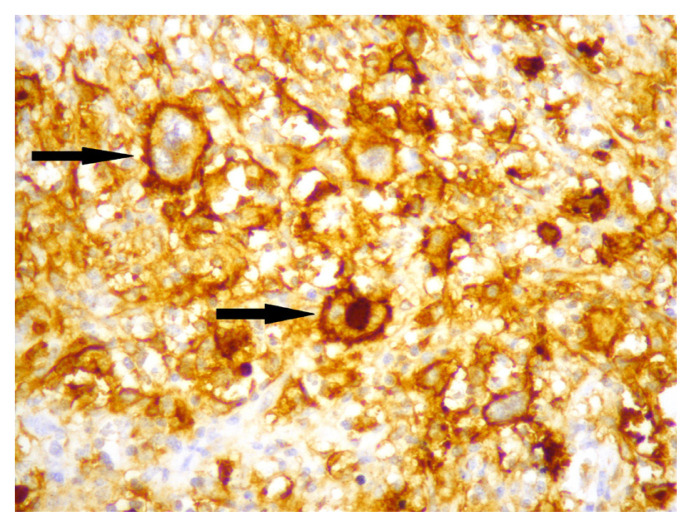
Strong (+3) membranous staining for PD-L1 in HRS cells (arrows) (×400).

**Figure 4:**
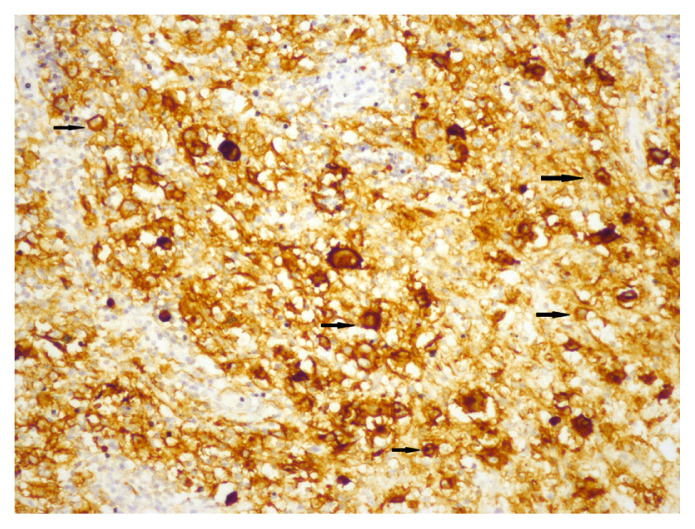
PD-L1 positivity in the tumour microenvironment (arrows) (×200).

**Figure 5:**
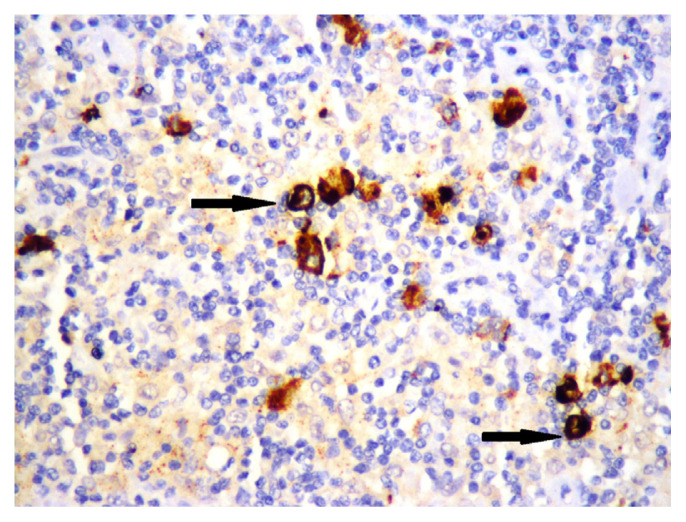
Membranous and cytoplasmic staining for EBV LMP1 in HRS cells (arrows) (×400).

Membranous staining for PD-L1 was evaluated via conventional light microscope in HRS cells and the cells of tumour microenvironment. In previous studies, similar criteria were used for staining characteristics and intensity, but different cut-off values were used to evaluate tumour cells and microenvironment [10–15]. We chose E1L3N clone, which has been validated with standard clones, for selective HRS staining [14]. We first used %5 PD-L1 staining as cut-off value for HRS cells and then evaluated the staining quality in three categories. Tumours with less than 5% of HRS cells stained with PD-L1 were categorized as negative (0), and then tumours which had PD-L1 staining in at least 5% of HRS cells were categorized qualitatively for staining intensity as weak (+1), moderate (+2), and strong (+3). Finally, patients with moderate (+2), and strong (+3) staining in at least 5% of HRS cells were considered “positive for PD-L1”, and others were considered “negative for PD-L1” (Figures 1–3). Tumours which had 20% or more PD-L1 staining in nonneoplastic cells were considered “positive tumour microenvironment” (Figure 4). Placental tissue was used for external positive control [10]. Membranous and cytoplasmic staining for EBV LMP1 in HRS cells was considered positive (Figure 5).

### 2.3. Ethical approval

All procedures performed in studies involving human participants were in accordance with the ethical standards of the institutional and/or national research committee and with the 1964 Helsinki Declaration and its later amendments or comparable ethical standards. The study was approved by the Bioethics Committee of Şişli Hamidiye Etfal Health Practice and Research Centre (No. 2579/2019).

### 2.4. Statistical analysis

Statistical analysis was performed with SPSS programme version 25.0 (IBM SPSS Statistics, USA). Descriptive statistics were number and percentage for categorical variables; median, minimum, and maximum for numerical variables. Comparisons of numerical variables in two independent groups were performed with the Mann–Whitney U test since normal distribution condition was not provided. The ratio of categorical variables between the groups was tested by chi-squared analysis. Monte Carlo simulation was applied when the conditions were not met. Statistical significance level was accepted as p < 0.05.

## 3. Results

### 3.1. Clinical and prognostic parameters

Thirty-nine patients were male (69.6%), 17 patients were (30.4%) female. Median age of the patients was 26 years; age interval was between 3 and 85 years. Cases were categorized for the histological subtypes as 25 (44.6%) nodular sclerosing subtype, 29 (51.8%) mixed cellularity subtype, 1 (1.8%) lymphocyte-rich subtype, and 1 (1.8%) lymphocyte-depleted subtype.

Clinical findings at the time of diagnosis could be obtained for 39 patients. Four of them (10.3%) were stage-1, 19 (48.7%) were stage-2, 9 (23.1%) were stage-3, and 7 (17.9%) were stage-4. Seven (17.9%) patients had splenic involvement, 4 (10.3%) patients had extranodal involvement, and 3 (7.3%) had bone marrow involvement. Twenty (51.3%) patients had B symptoms.Clinical follow-up of 34 patients showed that 27 (81.8%) were on remission, 1 (2.9%) was continuing therapy, 4 (12.1%) relapsed during follow-up, and 2 (5.9%) died from the disease (Table 1).

### 3.2. PD-L1 expression in CHL patients

Fifty (89.2%) of the cases had HRS cells “positive for PD-L1”. For nodular sclerosing subtype of CHL cases, 24 (96%) were positive for PD-L1. For mixed cellularity subtype CHL cases, 25 (86.2%) were positive for PD-L1. One case with lymphocyte-rich subtype of CHL was positive for PD-L1 and one case with lymphocyte-depleted subtype of CHL was negative for PD-L1. All cases had tumour microenvironment positive for PD-L1. There was no statistically significant difference among histological subtypes for PD-L1 positivity (p = 0.102) (Table 2).

### 3.3. EBV and PD-L1

EBV was positive in 29 patients (51.7%) (Table 1). Eight patients (32%) with nodular sclerosing subtype, 17 patients (68%) with mixed cellularity subtype, and one patient with lymphocyte-rich subtype were positive for EBV. One patient with lymphocyte-depleted subtype was negative for EBV. EBV positivity was statistically significantly higher on mixed cellularity subtype CHL compared to the remaining subtypes (p = 0.01). Mixed cellularity subtype CHL had significantly high EBV expression (Table 2).

Twenty-six out of 50 “PD-L1-positive” cases were EBV-positive and 3 out of 6 “PD-L1–negative” cases were EBV-positive. PD-L1 positivity did not show statistically significant difference according to EBV expression (p = 1.00) (Table 3).

### 3.4. PD-L1 expression and clinical parameters

Median patient age for PD-L1–positive cases was 25.5 years (7–80 years). PD-L1 was positive in 16 female and 34 male patients, and negative in 1 female and 5 male patients. Two of the four stage-1 patients were “positive for PD-L1”. PD-L1 was positive in all 19 stage-2 patients. Seven of the 9 stage-3 patients were “positive for PD-L1. Six of the 7 stage-4 patients were “positive for PD-L1”.

Six of 7 patients were positive for PD-L1 with splenic involvement, though 28 of 32 were also PD-L1–positive in uninvolved patients. Extranodal involvement was seen in 4 patients, all positive for PD-L1. Three patients had bone marrow involvement; all were positive for PD-L1. Eighteen of 20 PD-L1–positive patients had B symptoms. PD-L1 positivity did not show statistically significant difference according to patient age (p = 0.131), sex (p = 1.000), disease stage (p = 1.000), splenic involvement (p = 1.000), extralymphatic involvement (p = 1.000), bone marrow involvement (p = 1.000), and B symptoms (p = 0.661) (Table 4).

### 3.5. PD-L1 expression and prognosis

Prognostic data from 34 patients was collected from hospital records. 30 patients (88.2%) were “positive for PD-L1”. Median overall survival (OS) of all 34 cases was 24 (4–108) months and median disease-free survival (DFS) was 16 (0–96) months. For PD-L1–positive cases median OS was 23 (4–108) months and median DFS was 14 (0–96) months. For PD-L1–negative cases, median OS was 49 (8–61) months and median DFS was 37 (2–57) months.

PD-L1 was positive in 23 (79.3%) of 27 patients currently in remission. Recurrence was detected in 4 PD-L1–positive patients during follow-up. However, 25 of 29 nonrecurrent patients were also positive for PD-L1. Two PD-L1–positive patients died from the disease.

Prognostic factors did not show statistically significant difference according to PD-L1 positivity (Table 5).

## 4. Discussion

Our study revealed a high rate (89.2%) of PD-L1 positivity in HRS cells and all cases had tumour microenvironment positive for PD-L1. EBV expression was statistically significantly higher on mixed cellularity subtype. We did not find a statistically significant difference between PD-L1 positivity on HRS cells and EBV expression, clinical parameters, and prognosis.

PD-L1 on the surface of antigen presenting cells suppresses TCR production by stimulating PD1 on the surface of T cells. This process causes anergy in T cells [16,17]. In physiological conditions, it prevents tissue damage and autoimmune reactions by prolonged and excessive immune response via creating suppression in T cells [18]. Tumours expressing PD-L1 protein acquire the ability to escape cellular immunity by suppressing cytotoxic T cells. Therefore, the PD1/PDL1 pathway has been the target of newly emerging immunotherapy methods [19–21].

CHL is one of these tumours. PD-L1 expression has been observed on both malignant HRS cells and cells of tumour microenvironment [22,23]. Food and Drug Administration (FDA) has approved nivolumab and pembrolizumab, PD-1 inhibitors, in patients who do not respond to routine chemotherapy with disease relapse after bone marrow transplantation [8,9,19,24].

In our study, we found PD-L1 positivity in HRS cells in 50 (89.2%) of 56 cases. Although different results were obtained in various studies in the literature, increased PD-L1 expression (20%–95.7%) was detected in HRS cells in CHL. There are differences between the studies related to various reasons, such as clone and evaluation criteria and laboratory technique [10–15]. In our study, E1L3N clone, which shows selective staining in HRS cells and has been validated with standard clones, was used; both staining intensity and prevalence were evaluated [13,14,25–27].

We found 89.2% PD-L1 positivity in CHL. It was like those found by Chen et al. [10], Sakakibara et al. [12], Koh et al. [13], Menter et al. [14], Inaguma et al. [15], Dilly-Feldis et al. [28], and Gerhard-Hartmann et al. [29], but it was considerably higher than the result of Paydaş et al. (20%) [11], Jimenez et al. (44%) [30], and Tanaka et al. (62%) [31]. The results may differ due to PD-L1 clone and evaluation criteria regarding staining quality and intensity.

Wei Xing et al. studied with E1L3N clone in bone marrow of 44 CHL cases and diagnostic tissue biopsy from 30 of them. They found 8 cases had bone marrow involvement by CHL. All 8 of them had either 3+ or 2+ membranous staining for PD-L1. Moreover, all 30 of the nonbone marrow diagnostic tissue had PD-L1 expression in HRS and tumour microenvironment [32].

Fluorescence in situ hybridization (FISH) for PD-L1 was used besides immunohistochemistry. Roemer et al. found polysomia, copy number enhancement, or amplification at the PD-L1 gene locus in 97% of their cases, which was correlated with immunohistochemical results [33]. Likewise, a study on patients of the German Hodgkin Study Group NIVAHL trial showed copy number alterations on PD-L1 locus in all specimens with variable severity and 97% PD-L1 expression on immunohistochemistry [29]. However, Jimenez et al. found genetic alterations in only 38% of paediatric CHL cases by FISH and 44% by immunohistochemistry [30]. However, FISH and immunohistochemistry results were compatible in all these studies.

Sanger sequence method was used in one study on 40 paediatric CHL patients. Of the patients, 20.5% had p.R260C and 7.7% had p.R234L mutations on exon 5 of PD-L1 locus [34]. In a small-scale next generation sequencing (NGS) study performed on 4 CHL patients who were positive for PD-L1 with immunohistochemistry, despite 3 of the patients were PD-L1 amplified by FISH, none were amplified by NGS method [35]. Plasma levels of PD-L1 in CHL cases were elevated but it was not correlated with tissue expression by immunohistochemistry [36,37].

PD-L1 is expressed not only in neoplastic cells but also by macrophages, lymphocytes, neutrophils, and fibroblasts in the tumour microenvironment. Increased PD-L1 expression has been reported in background macrophages in CHL cases [10–12]. Hollander et al. found significant correlation between unfavourable prognosis and PD-L1 expression in the tumour microenvironment but they did not find any correlation with PD-L1 expression on HRS cells [38]. We observed PD-L1 positivity in macrophages forming tumour microenvironment in all cases. Although this makes it difficult to evaluate HRS, we observed HRS cells staining more intense than the reactive cells in the tumour background with E1L3N clone.

EBV positivity has been reported in approximately 20%–100% of CHL. Among the histological subtypes, it was less common with nodular sclerosing subtype of CHL than mixed cellularity subtype of CHL [5,6]. Studies have shown that EBV can activate the PD-L1/PD-L2 gene locus on 9p24.1 [39]. In our study, 29 (51.9%) of the cases were found to have EBV positivity. It was significantly higher in patients with mixed cellularity subtype of CHL than patients with nodular sclerosing subtype of CHL (p = 0.010).

We compared PD-L1 positivity in EBV-positive and -negative cases. PD-L1 positivity was similar in both groups. Although there have been reports that the presence of EBV correlates with PD-L1 expression [39], we found no statistically significant relationship between them. Other studies also support our results [12,13,15,40]. Publications that show correlation are mainly based on the correlation between EBV positivity and aberrations or promoter activation at the PD-L1 gene locus. However, no such association was found in studies comparing EBV and PD-L1 expression at protein level. This suggests that other pathways leading to PD-L1 activation may also be present in EBV-negative cases [41].

The effect of PD-L1 expression on clinical findings was examined in various studies, and no correlation was found [11,13,14]. However, in some studies, patients with 9p24.1 amplification showed advanced clinical stage and shorter disease-free survival [33]. Sakakibara et al. performed a study with SP142 clone, and they found lower PD-L1 positivity in lymphocyte-rich subtype of CHL cases [12]. Gül et al. found significant correlation between pR260c mutation on exon 5 of PD-L1 gene and nodular sclerosing subtype and event-free survival in older paediatric patients. This may be due to decreased functionality in the mutant protein [34]. In our study, clinical data of 39 patients were examined, no significant relationship was found between PD-L1 positivity and age, sex, histological subtype of CHL, clinical stage, presence of spleen, bone marrow, or other extranodal organ involvement at the time of diagnosis.

We reached follow-up records of 34 patients. We did not find any difference between PD-L1–positive and -negative cases in terms of total survival and disease-free survival. Total follow-up period was 24 months (4–108 months). In the study of Koh et al., there was no relationship between PD-L1 expression and overall survival in 109 patients. The median follow-up period was 4.91 years (0.17–17.33 years) [13].

In our study, disease recurred in 4 cases, and 2 patients died from disease. PDL1 was positive in all these cases. However, this was not statistically significant. Paydaş et al. found overall and disease-free survival in patients expressing both PD1 and PD-L1 were significantly lower [11]. Koh et al. reported that PD1-positive patients had lower 5-year survival [13].

We found high rate of PD-L1 positivity in HRS cells, and in APCs of the tumour microenvironment, but without any correlation with EBV expression, clinical findings, and prognosis. Despite the lack of clinical correlation and PD-L1 expression of the tumour, the high prevalence of PD-L1 positivity in HRS cells and APCs gives hope for new therapeutic possibilities targeting the programmed death pathway in these patients because although modern therapeutic protocols have high success rates, an important portion of the patients still cannot achieve complete remission or relapses still occur.

Due to high PD-L1 expression rate, the positive and negative groups were not evenly distributed. This caused a bias when we compared positive and negative patients for clinical parameters and prognosis. Another limitation was that we could not reach the clinical data of all patients.

## Figures and Tables

**Table 1:** Descriptive statistics.

		n	%
Sex	Female	17	30.4
	Male	39	69.6
Histological subtype	Nodular sclerosing	25	44.6
	Mixed cellularity	29	51.8
	Lymphocyte-rich	1	1.8
	Lymphocyte-depleted	1	1.8
Stage	Stage 1	4	10.3
	Stage 2	19	48.7
	Stage 3	9	23.1
	Stage 4	7	17.9
Splenic involvement	Positive	7	17.9
	Negative	32	82.1
Extralymphatic involvement	Positive	4	10.3
	Negative	35	89.7
Bone marrow involvement	Positive	3	7.3
	Negative	38	92.7
B symptoms	Positive	20	51.3
	Negative	19	48.7
EBV	Positive	29	51.8
	Negative	27	48.2
Remission	Positive	27	81.8
	Negative	6	18.2
Recurrence	Positive	4	12.1
	Negative	29	87.9
Death from disease	Positive	2	5.9
	Negative	32	94.1

**Table 2:** PD-L1 and EBV positivity rates according to histological subtypes.

		PDL1					EBV				
		Negative		Positive			Negative		Positive		
		n	%	n	%	p	n	%	n	%	p
Histological subtype	NSCHL	1	16.7	24	48.0	0.120	17	68	8	32	0.010
	MCCHL	4	66.6	25	50.0		9	31	20	69	
	LRCHL	0	0.0	1	2.0		0	0.0	1	100	
	LDCHL	1	16.7	0	0.0		1	100	0	0.0	

(NSCHL: Nodular sclerosing subtype, MCCHL: Mixed cellular type, LRCHL: Lymphocyte-rich subtype, LPCHL: Lymphocyte-depleted subtype)

**Table 3:** Comparison of PD-L1 positivity according to EBV positivity.

	PD-L1					
	Negative			Positive		
		n	%	n	%	p
EBV expression	Negative	3	50.0	24	48.0	1.000
	Positive	3	50.0	26	52.0	

**Table 4:** Comparison of PD-L1 positivity on HRS cells according to clinical data at the time of diagnosis.

		PDL1				
		Negative		Positive		
		Median	Min–max	Median	Min–max	p
Age (years)		56	7–80	25.5	3–85	0.131
		n	%	n	%	p
Sex	Female	1	16.7	16	32.0	0.655
	Male	5	83.3	34	68.0	
Stage	Stage 1	2	40.0	2	5.9	1.000
	Stage 2	0	0.0	19	55.9	
	Stage 3	2	40.0	7	20.6	
	Stage 4	1	20.0	6	17.6	
Splenic involvement	Negative	4	80.0	28	82.4	1.000
	Positive	1	20.0	6	17.6	
Extralymphatic involvement	Negative	5	100	30	88.2	1.000
	Positive	0	0.0	4	11.8	
Bone marrow involvement	Negative	5	100	33	91.7	1.000
	Positive	0	0.0	3	8.3	
B symptoms	Negative	3	60.0	16	47.1	0.661
	Positive	2	40.0	18	52.9	

**Table 5:** Comparison of prognosis in PD-L1–positive and PD-L1–negative cases.

		PD-L1–negative		PD-L1–positive		
		Median	Min–max	Median	Min–max	p
Overall survival (month)		49	8–61	23	4–108	0.504
Disease-free survival (month)		37	2–57	14	0–96	0.538
		n	%	n	%	p
Remission	Positive	4	100	23	79.3	1.000
	Negative	0	0.0	6	20.7	
Recurrence	Positive	0	0.0	4	13.8	1.000
	Negative	4	100	25	86.2	
Death	Positive	0	0.0	2	6.7	1.000
	Negative	4	100	28	93.3	
